# Hypercalcemia Secondary to Immune Reconstitution Inflammatory Syndrome in an HIV-Infected Individual With Mycobacterium avium Complex

**DOI:** 10.7759/cureus.18174

**Published:** 2021-09-21

**Authors:** Sanjana S Awasty, Sabih Jafri, Saima Manzoor, Abid Yaqub

**Affiliations:** 1 Division of Internal Medicine, University of Cincinnati Medical Center, Cincinnati, USA; 2 Division of Endocrinology, Diabetes and Metabolism, University of Cincinnati Medical Center, Cincinnati, USA; 3 Division of Endocrinology, Diabetes and Metabolism, University of Cincinnati, College of Medicine, Cincinnati, USA

**Keywords:** haart, hiv aids, mycobacterium avium intracellulare, immune reconstitution syndrome, acute hypercalcemia

## Abstract

Immune reconstitution inflammatory syndrome (IRIS) is an uncommon cause of hypercalcemia in HIV-infected patients recently started on highly active antiretroviral therapy (HAART). It is hypothesized that increased granulomatous formation due to IRIS leads to an overproduction of calcitriol. High levels of calcitriol, then, can lead to significant hypercalcemia. We present the case of a 63-year-old male with HIV off HAART presented to the emergency room for confusion, frequent falls, and cough. His CD-4 count was noted to be below 35 cells/µL (255-2,496). Over the course of the hospitalization, the patient was found to have disseminated *Mycobacterium avium c*omplex (MAC) infection and was initiated on HAART. Initiation of HAART was followed by an increase in calcium up to 14.1 mg/dL. The hypercalcemia did not respond to either Calcitonin or Pamidronate. Consideration was then given to IRIS in the setting of MAC infection leading to increased granulomatous formation. Calcium levels normalized within three days of therapy after initiation of prednisone for the treatment of IRIS.  It is thought that an increase in CD-4 counts leads to the recovery of an immune response. This can lead to granulomatous inflammation. An increase in granuloma formation can cause hypercalcemia due to overproduction of calcitriol via increased 1𝛼-hydroxylase activity from macrophages. Our case report describes IRIS-mediated hypercalcemia in an HIV-infected individual with MAC infection. This unusual cause of severe hypercalcemia should be considered in differential diagnoses for immunocompromised patients in the appropriate setting. Prompt treatment of IRIS with glucocorticoids can lead to the resolution of hypercalcemia.

## Introduction

Severe hypercalcemia (>14 mg/dL) typically necessitates investigation for underlying malignancy, though it is important to also complete a thorough evaluation to rule out other causes of hypercalcemia. The commonest cause of hypercalcemia presenting in an outpatient setting is primary hyperparathyroidism whereas malignancy-related hypercalcemia is the most prevalent cause of significant symptomatic hypercalcemia in the hospitalized setting [1]. One unusual etiology of hypercalcemia is immune reconstitution inflammatory syndrome (IRIS). IRIS describes the phenomenon of paradoxical clinical deterioration observed in patients who are started on therapy for systemic disease. It typically occurs in HIV-infected individuals who are started on highly active antiretroviral therapy (HAART) [2] though it has also been seen in patients being treated for *Mycobacterium tuberculosis *[3]. Interestingly, the syndrome occurs despite expected decreases in HIV viral load and increases in CD4 counts after initiation of therapy. Two types of IRIS have been noted in the literature. “Paradoxical IRIS” refers to the worsening of a pre-existing infection while “unmasking IRIS” describes the initial revelation of a previously unknown pre-existing infection. It typically occurs within 60 days of starting antiretroviral therapy [4]. It has been hypothesized that IRIS-mediated granulomatous formation can cause hypercalcemia due to overproduction of calcitriol via an increase in 1𝛼-hydroxylase activity [5]. Below, we present a case of IRIS-mediated hypercalcemia in the setting of *Mycobacterium avium* complex (MAC) infection.

## Case presentation

A 63-year-old male with a past medical history significant for dementia and HIV presented to the emergency room with confusion, frequent falls, and productive cough. He was found on the floor by his mother on the day of the presentation. On arrival to the emergency room, he was found to be tachycardic with a heart rate of 109, blood pressure of 133/89, and respiratory rate of 26, saturating 98% on room air. His physical exam was significant for a 5 cm x 5 cm area of ecchymoses over the right parietal scalp and chest wall tenderness to palpation. He was alert and oriented to place, time, and self and was moving all of his extremities spontaneously without overt abnormal neurological findings. He stated that he was diagnosed with HIV in 2014. His HIV risk factors included sexual encounters with multiple female and male partners. He took efavirenz-emtricitabine-tenofovir from 2014 to 2015 but then self-discontinued the therapy as he had to travel for work.  He was not able to provide additional information about his HIV. He was found to be anemic with a hemoglobin of 8.5 g/dL (13.2-17.1) and had an elevated serum creatinine kinase of 856 µ/L (30-223). During workup, the patient was noted to have a CD-4 count of <35 cells/µL (255-2496), viral load of 658,000 copies/mL (undetectable), and corrected calcium of 11 mg/dL (8.6-10.3).

**Figure 1:**
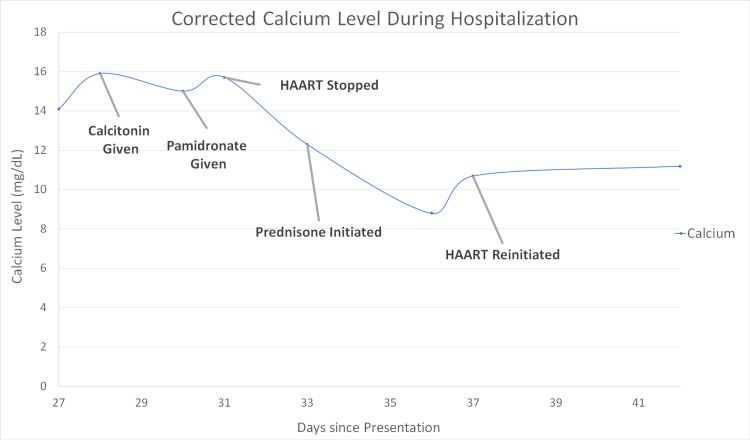
Corrected calcium levels during hospitalization. Day 28 calcitonin given. Day 30 pamidronate given. Day 31 HAART stopped. Day 33 prednisone 60 mg initiated. Day 37 HAART reinitiated. HAART - highly active antiretroviral therapy

Infectious disease was consulted for productive cough with abnormal CT imaging findings in the setting of a new diagnosis of AIDS (given CD-4 count <200 cells/µL). Sputum cultures were obtained on day 4 and day 6 of hospitalization which grew *Mycobacterium avium*. He was started on HAART therapy with bictegravir, emtricitabine, tenofovir, and alafenamide regimen on day 8. On day 27, the patient’s corrected calcium was noted to suddenly increase to 14.1 mg/dL. Further testing revealed a TSH of 1.18 IU/mL (0.45-4.12), PTH 4.0 pg/mL (12-88), and a 25-hydroxy vitamin D level of 54.1 ng/mL (30-100). He was given calcitonin 280 IU twice a day subcutaneously for hypercalcemia. MAC treatment was initiated with rifabutin, ethambutol, clarithromycin, and azithromycin. In addition, his HAART therapy was switched to emtricitabine, tenofovir, alafenamide, and dolutegravir given concern for medication interactions. Despite these interventions, no improvement in hypercalcemia was noted. Therefore, endocrinology was consulted on day 30 to assist with the management of hypercalcemia. On day 30, IV Pamidronate 90 mg was given but the patient’s hypercalcemia persisted (Figure 1). On day 31, consideration was given to the possibility of IRIS-induced hypercalcemia in the setting of a patient with an underlying MAC infection. HAART therapy was, thus, discontinued at that time. He was ultimately found to have a negative PTH-rP and elevated calcitriol of 115 pg/mL (19.9-79.3). On day 33, he was initiated on prednisone 60 mg to treat IRIS and was noted to have normalized corrected calcium of 8.8 mg/dL by day 36. The prednisone was initiated at 60 mg for five days then tapered to 40 mg for five days, 20 mg for five days, followed by 10 mg for five days. HAART was reinitiated on day 37 after resolution of hypercalcemia and calcium levels remained stable thereafter.

## Discussion

Hypercalcemia is a relatively common problem encountered in clinical practice. Our case highlights an unusual cause of hypercalcemia: granuloma-induced hypercalcemia in the setting of IRIS. There are no universally accepted diagnostic criteria for IRIS though it generally includes the presence of a low CD-4 count, initiation of HAART, and presentation of symptoms suggesting an inflammatory state. The exact mechanism of IRIS is unknown though it has been postulated that the increase in CD-4 counts leads to recovery of immune response to specific antigens which may lead to IRIS [2,6]. This, in turn, can lead to granulomatous inflammation [2]. An increase in granuloma formation can cause hypercalcemia due to overproduction of calcitriol via increased 1𝛼-hydroxylase activity from macrophages [5]. This observation has been noted in isolated case reports in HIV-infected individuals with a variety of infectious and noninfectious processes including mycobacterium [3,7-10], cryptococcal [11], and lymphoma [12].

**Figure 2:**
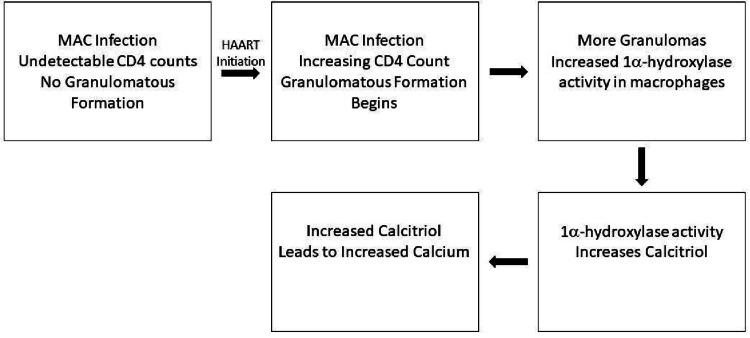
Suggested pathogenesis of IRIS leading to hypercalcemia in the setting of MAC infection. HAART - highly active antiretroviral therapy; IRIS - Immune reconstitution inflammatory syndrome; MAC - Mycobacterium avium complex

Interestingly, this presentation did not occur until initial treatment with HAART therapy leading to new granulomatous inflammation (Figure 2). The high serum calcitriol level further supported the idea that hypercalcemia was related to a granulomatous process due to increased conversion of 25-hydroxy Vitamin D to the active form of 1, 25-dihydroxy vitamin D [13]. Treatment of IRIS is challenging as there is sparse evidence-based data on effective treatments. Meintjes et al. demonstrated, in a randomized control trial, that a four-week trial of prednisone (1.5 mg/kg/day for two weeks then 0.75 mg/kg/day for two weeks) reduced the need for hospitalization, decreased serum inflammatory marker levels, and improved quality of life [14]. Unfortunately, to the best of our knowledge, there are no established guidelines on the management of hypercalcemia in the setting of IRIS due to the rare nature of the condition, difficulty in diagnosis of IRS, and lack of published data regarding effective treatment options. Some case reports, though, have suggested that initiation of high-dose prednisone along with treatment for an underlying infection as well as continuing antiretroviral therapy are sufficient to manage severe hypercalcemia [15]. This management regimen is further supported by this case report as there was a noted plateauing of the serum calcium levels after initiation of prednisone in the setting of treatment for MAC. Furthermore, calcium levels remained stable on day 53, 23 days after pamidronate was given and outside its’ duration of action lending itself to the notion that treatment of IRIS was essential in the treatment of this patient’s hypercalcemia. There is no consensus about the optimal timing of initiating antiretroviral therapy after treatment of an opportunistic infection (OI) though most guidelines recommend beginning therapy within two weeks of treatment for an OI [16].

## Conclusions

IRIS-mediated hypercalcemia is a rare adverse event that is observed after starting antiretroviral therapy. Management includes the initiation of high-dose prednisone and treatment of any known opportunistic infections. Given the variety of infections patients with untreated HIV may have that also present with granulomas such as histoplasmosis, candidiasis, *Pneumocystis carinii* pneumonia, etc., consideration should be given to IRIS-mediated hypercalcemia in those with the recent initiation of HAART therapy.
